# Associations Between Neighborhood Environments and Depressive Symptoms of Older Adults

**DOI:** 10.1093/geroni/igab046.1809

**Published:** 2021-12-17

**Authors:** Yuqi Liu, Shiyu Lu, Yingqi Guo, Hung Chak Ho, Hiu Kwan Chui, Chris Webster, Lai Har Chiu, Terry Y S Lum

**Affiliations:** The University of Hong Kong, Hong Kong, Hong Kong

## Abstract

Little is known about the accumulative impacts of neighbourhood physical environments on depression among older adults. Based on a cohort study of 2,081 older adults in Hong Kong, this study examined longitudinal relationships between neighbourhood physical environments and depressive symptoms among older adults and the moderating effects of the slope of terrain and individual functional ability using latent growth curve modelling. Results indicated that the availability of community centres and passive leisure facilities reduced depressive symptoms over time. The protective effects of residential surrounding greenness on depressive symptoms among older adults differed by the slope of terrain. Longitudinal associations between neighbourhood physical environments and depressive symptoms varied between older adults with and without functional limitations. Identifying environmental barriers and applying targeted residential environment interventions are essential.

